# Nosology and Classification of Genetic Skeletal Disorders: 2010 Revision

**DOI:** 10.1002/ajmg.a.33909

**Published:** 2011-03-15

**Authors:** Matthew L Warman, Valerie Cormier-Daire, Christine Hall, Deborah Krakow, Ralph Lachman, Martine LeMerrer, Geert Mortier, Stefan Mundlos, Gen Nishimura, David L Rimoin, Stephen Robertson, Ravi Savarirayan, David Sillence, Juergen Spranger, Sheila Unger, Bernhard Zabel, Andrea Superti-Furga

**Affiliations:** 1 Orthopaedic Research Laboratories, Department of Orthopaedic Surgery, The Howard Hughes Medical Institute, Children's HospitalBoston, Massachusetts; 2 Department of Genetics and INSERM U781, Paris Descartes University, Hôpital Necker Enfants MaladesParis, France; 3 Institute of Child Health, University of LondonLondon, UK; 4 Medical Genetics Institute, Steven Spielberg Building, Cedars-Sinai Medical CenterLos Angeles, California; 5 Departments of Orthopaedic Surgery and Human Genetics, UCLALos Angeles, California; 6 Department of Medical Genetics, University Hospital of Antwerp, University of AntwerpEdegem, Belgium; 7 Institut für Medizinische Genetik, Charité Universitätsmedizin Berlin, Max-Planck-Institut für Molekulare GenetikBerlin, Germany; 8 Department of Pediatric Imaging, Tokyo Metropolitan Children's Medical CenterFuchu, Tokyo, Japan; 9 Department of Paediatrics and Child Health, Dunedin School of Medicine, Otago UniversityDunedin, New Zealand; 10 Murdoch Children's Research Institute, Royal Children's Hospital, Department of Paediatrics, University of MelbourneVictoria, Australia; 11 Discipline of Genetic Medicine, The Children's Hospital at Westmead Clinical School, The University of SydneyWestmead, Australia; 12 Centre for Pediatrics and Adolescent Medicine, Freiburg University Hospital, University of FreiburgFreiburg, Germany; 13 Medical Genetics Service, University of Lausanne, CHUV—Centre Hospitalier Universitaire VaudoisLausanne, Switzerland; 14 Department of Pediatrics, University of Lausanne, CHUV—Centre Hospitalier Universitaire VaudoisLausanne, Switzerland

**Keywords:** skeletal genetics, osteochondrodysplasias, nosology, dysostoses, molecular basis of disease

## Abstract

Genetic disorders involving the skeletal system arise through disturbances in the complex processes of skeletal development, growth and homeostasis and remain a diagnostic challenge because of their variety. The Nosology and Classification of Genetic Skeletal Disorders provides an overview of recognized diagnostic entities and groups them by clinical and radiographic features and molecular pathogenesis. The aim is to provide the Genetics, Pediatrics and Radiology community with a list of recognized genetic skeletal disorders that can be of help in the diagnosis of individual cases, in the delineation of novel disorders, and in building bridges between clinicians and scientists interested in skeletal biology. In the 2010 revision, 456 conditions were included and placed in 40 groups defined by molecular, biochemical, and/or radiographic criteria. Of these conditions, 316 were associated with mutations in one or more of 226 different genes, ranging from common, recurrent mutations to “private” found in single families or individuals. Thus, the Nosology is a hybrid between a list of clinically defined disorders, waiting for molecular clarification, and an annotated database documenting the phenotypic spectrum produced by mutations in a given gene. The Nosology should be useful for the diagnosis of patients with genetic skeletal diseases, particularly in view of the information flood expected with the novel sequencing technologies; in the delineation of clinical entities and novel disorders, by providing an overview of established nosologic entities; and for scientists looking for the clinical correlates of genes, proteins and pathways involved in skeletal biology. © 2011 Wiley-Liss, Inc.

## INTRODUCTION

In the 1960s, accumulating evidence that genetic skeletal disorders were clinically and genetically heterogeneous prompted a group of international experts to prepare a document to reach an agreement on the nomenclature of what was then called “constitutional (or intrinsic) disorders of bone” [1970, 1971a,b,c,d; McKusick and Scott, 1971]. The “Nomenclature” was meant to bring together experts in radiology, clinical genetics, and pediatrics to agree on the denomination and classification of skeletal disorders, syndromes and metabolic diseases that were being newly described. Revisions have been prepared in 1977, 1983, 1992, and 1997 [1978, 1979, 1983, 1998, Rimoin, 1979; Spranger, 1992; Lachman, 1998]. Following the establishment of the International Skeletal Dysplasia Society (ISDS) in 1999, and to cope with the increasing complexity of information, revisions of the Nosology have been delegated to an expert group nominated ad hoc within the ISDS to ensure an adequate representation of clinical, radiological and molecular expertise (2001 and 2006 revisions) [Hall, 2002; Superti-Furga and Unger, 2007].

## METHODS

The Nosology Group of the International Skeletal Dysplasia Society met in August 2009. A consensus was reached for changes to be made to the grouping of disorders and about the inclusion of individual disorders. The drafts were circulated after the meeting and an effort was made to monitor recent publications up to November 2010. The criteria used for inclusion of individual disorders were unchanged from the previous revision. They were:

1. Significant skeletal involvement, corresponding to the definition of skeletal dysplasias, metabolic bone disorders, dysostoses, and skeletal malformation and/or reduction syndromes.
2. Publication and/or listing in MIM (meaning that observations should not find their way into the Nosology before they achieve peer-reviewed publication status).
3. Genetic basis proven by pedigree or very likely based on homogeneity of phenotype in unrelated families.
4. Nosologic autonomy confirmed by molecular or linkage analysis and/or by the presence of distinctive diagnostic features and of observation in multiple individuals or families.

## RESULTS

Four hundred fifty-six different conditions were included and placed in 40 groups defined by molecular, biochemical and/or radiographic criteria. Of these conditions, 316 (*2006 revision*: 215) were associated with one or more of 226 (*2006 revision*: 140) different genes. The results are presented in Table I. Within a group, disorders with known molecular basis have been listed preceding those with lesser degree of evidence; however, variants of the same disorder have been kept together.

**TABLE I:** 

Group/name of disorder	Inheritance	MIM No.	Locus	Gene	Protein	Notes
**1. FGFR3 chondrodysplasia group**						
Thanatophoric dysplasia type 1 (TD1)	AD	187600	4p16.3	*FGFR3*	FGFR3	Includes previous San Diego type
Thanatophoric dysplasia type 2 (TD2)	AD	187601	4p16.3	*FGFR3*	FGFR3	
Severe achondroplasia with developmental delay and acanthosis nigricans (SADDAN)	AD	See 187600	4p16.3	*FGFR3*	FGFR3	
Achondroplasia	AD	100800	4p16.3	*FGFR3*	FGFR3	
Hypochondroplasia	AD	146000	4p16.3	*FGFR3*	FGFR3	
Camptodactyly, tall stature, and hearing loss syndrome (CATSHL)	AD	187600	4p16.3	*FGFR3*	FGFR3	Inactivating mutation
Hypochondroplasia-like dysplasia(s)	AD, SP					Similar to hypochondroplasia but unlinked to FGFR3, probably heterogeneous; uncertain diagnostic criteria
*See also group 33 for craniosynostoses syndromes linked to FGFR3 mutations, as well as LADD syndrome in group 39 for another FGFR3-related phenotype*						
**2. Type 2 collagen group and similar disorders**						
Achondrogenesis type 2 (ACG2; Langer–Saldino)	AD	200610	12q13.1	*COL2A1*	Type 2 collagen	
Platyspondylic dysplasia, Torrance type	AD	151210	12q13.1	*COL2A1*	Type 2 collagen	See also severe spondylodysplastic dysplasias (group 13)
Hypochondrogenesis	AD	200610	12q13.1	*COL2A1*	Type 2 collagen	
Spondyloepiphyseal dysplasia congenita (SEDC)	AD	183900	12q13.1	*COL2A1*	Type 2 collagen	
Spondyloepimetaphyseal dysplasia (SEMD) Strudwick type	AD	184250	12q13.1	*COL2A1*	Type 2 collagen	
Kniest dysplasia	AD	156550	12q13.1	*COL2A1*	Type 2 collagen	
Spondyloperipheral dysplasia	AD	271700	12q13.1	*COL2A1*	Type 2 collagen	
Mild SED with premature onset arthrosis	AD		12q13.1	*COL2A1*	Type 2 collagen	Often associated with p.R719C and p.G474S mutations
SED with metatarsal shortening (formerly Czech dysplasia)	AD	609162	12q13.1	*COL2A1*	Type 2 collagen	Often associated with the p.R275C mutation
Stickler syndrome type 1	AD	108300	12q13.1	*COL2A1*	Type 2 collagen	
Stickler-like syndrome(s)						Unlinked to either COL2A1, COL11A1, or COL11A2. See also COL9A1 for recessive form
**3. Type 11 collagen group**						
Stickler syndrome type 2	AD	604841	1p21	*COL11A1*	Type 11 collagen alpha-1 chain	
Marshall syndrome	AD	154780	1p21	*COL11A1*	Type 11 collagen alpha-1 chain	
Fibrochondrogenesis	AR	228520	1p21	*COL11A1*	Type 11 collagen alpha-1 chain	
Otospondylomegaepiphyseal dysplasia (OSMED), recessive type	AR	215150	6p21.3	*COL11A2*	Type 11 collagen alpha-2 chain	
Otospondylomegaepiphyseal dysplasia (OSMED), dominant type (Weissenbacher–Zweymüller syndrome, Stickler syndrome type 3)	AD	215150	6p21.3	*COL11A2*	Type 11 collagen alpha-2 chain	
*See also Stickler syndrome type 1 in group 2*						
**4. Sulfation disorders group**						
Achondrogenesis type 1B (ACG1B)	AR	600972	5q32–33	*DTDST*	SLC26A2 sulfate transporter	Formerly known as Fraccaro type achondrogenesis
Atelosteogenesis type 2 (AO2)	AR	256050	5q32–33	*DTDST*	SLC26A2 sulfate transporter	Includes de la Chapelle dysplasia, McAlister dysplasia, and “neonatal osseous dysplasia”
Diastrophic dysplasia (DTD)	AR	222600	5q32–33	*DTDST*	SLC26A2 sulfate transporter	
MED, autosomal recessive type (rMED; EDM4)	AR	226900	5q32–33	*DTDST*	SLC26A2 sulfate transporter	See also multiple epiphyseal dysplasias and pseudoachondroplasia group (group 9)
SEMD, PAPSS2 type	AR	603005	10q23–q24	*PAPSS2*	PAPS-Synthetase 2	Formerly “Pakistani type.” See also SEMD group (group 11)
Chondrodysplasia with congenital joint dislocations, CHST3 type (recessive Larsen syndrome)	AR	608637	10q22.1	*CHST3*	Carbohydrate sulfotransferase 3; chondroitin 6-sulfotransferase	Includes recessive Larsen syndrome, humero-spinal dysostosis, and SED Omani type
Ehlers–Danlos syndrome, CHST14 type (“musculo-skeletal variant”)	AR	601776	15q14	*CHST14*	Carbohydrate sulfotransferase 14; dermatan 4-sulfotransferase	Includes Adducted Thumb–Clubfoot syndrome
*See also group 7 and group 26 for other conditions with multiple dislocations*						
**5. Perlecan group**						
Dyssegmental dysplasia, Silverman-Handmaker type	AR	224410	1q36–34	*PLC* (*HSPG2*)	Perlecan	
Dyssegmental dysplasia, Rolland-Desbuquois type	AR	224400	1q36–34	*PLC* (*HSPG2*)	Perlecan	
Schwartz–Jampel syndrome (myotonic chondrodystrophy)	AR	255800	1q36–34	*PLC* (*HSPG2*)	Perlecan	Mild and severe forms; includes previous Burton dysplasia
**6. Aggrecan group**						
SED, Kimberley type	AD	608361	15q26	*AGC1*	Aggrecan	
SEMD, Aggrecan type	AR	612813	15q26	*AGC1*	Aggrecan	
Familial osteochondritis dissecans	AD	165800	15q26	*AGC1*	Aggrecan	
**7. Filamin group and related disorders**						
Frontometaphyseal dysplasia	XLD	305620	Xq28	*FLNA*	Filamin A	Some cases apparently lack FLNA mutations
Osteodysplasty Melnick–Needles	XLD	309350	Xq28	*FLNA*	Filamin A	
Otopalatodigital syndrome type 1 (OPD1)	XLD	311300	Xq28	*FLNA*	Filamin A	
Otopalatodigital syndrome type 2 (OPD2)	XLD	304120	Xq28	*FLNA*	Filamin A	
Terminal osseous dysplasia with pigmentary defects (TODPD)	XLD	300244	Xq28	*FLNA*	Filamin A	
Atelosteogenesis type 1 (AO1)	AD	108720	3p14.3	*FLNB*	Filamin B	Includes Boomerang dysplasia, Piepkorn dysplasia, and spondylohumerofemoral (giant cell) dysplasia
Atelosteogenesis type 3 (AO3)	AD	108721	3p14.3	*FLNB*	Filamin B	
Larsen syndrome (dominant)	AD	150250	3p14.3	*FLNB*	Filamin B	
Spondylo-carpal-tarsal dysplasia	AR	272460	3p14.3	*FLNB*	Filamin B	
Spondylo-carpal-tarsal dysplasia	AR	272460				Unlinked to FLNB
Franck–ter Haar syndrome	AR	249420	5q35.1	*SH3PXD2B*	TKS4	
Serpentine fibula—polycystic kidney syndrome	AD?	600330				
*See also group 4 for recessive Larsen syndrome and group 26 for conditions with multiple dislocations*						
**8. TRPV4 group**						
Metatropic dysplasia	AD	156530	12q24.1	*TRPV4*	Transient receptor potential cation channel, subfamily V, member 4	Includes lethal and non-lethal forms
Spondyloepimetaphyseal dysplasia, Maroteaux type (Pseudo–Morquio syndrome type 2)	AD	184095	12q24.1	*TRPV4*	Transient receptor potential cation channel, subfamily V, member 4	
Spondylometaphyseal dysplasia, Kozlowski type	AD	184252	12q24.1	*TRPV4*	Transient receptor potential cation channel, subfamily V, member 4	
Brachyolmia, autosomal dominant type	AD	113500	12q24.1	*TRPV4*	Transient receptor potential cation channel, subfamily V, member 4	
Familial digital arthropathy with brachydactyly	AD	606835	12q24.1	*TRPV4*	Transient receptor potential cation channel, subfamily V, member 4	
**9. Short-ribs dysplasias (with or without polydactyly) group**						
Chondroectodermal dysplasia (Ellis–van Creveld)	AR	225500	4p16	*EVC1*	EvC gene 1	
			4p16	*EVC2*	EvC gene 2	
Short rib—polydactyly syndrome (SRPS) type 1/3 (Saldino-Noonan/Verma-Naumoff)	AR	263510	11q22.3	*DYNC2H1*	Dynein, cytoplasmic 2, heavy chain 1	
SRPS type 1/3 (Saldino-Noonan/Verma-Naumoff)	AR	263510	3q25.33	*IFT80*	Intraflagellar transport 80 (homolog of)	
SRPS type 1/3 (Saldino-Noonan/Verma-Naumoff)	AR	263510				Unlinked to either DYNC2H1 or IFT80
SRPS type 2 (Majewski)	AR	263520		*NEK1*	Nima related kinase 1	
SRPS type 4 (Beemer)	AR	269860				
Oral-facial-digital syndrome type 4 (Mohr–Majewski)	AR	258860				
Asphyxiating thoracic dysplasia (ATD; Jeune)	AR	208500	3q25.33	*IFT80*	Intraflagellar transport 80 (homolog of)	
Asphyxiating thoracic dysplasia (ATD; Jeune)	AR	208500	11q22.3	*DYNC2H1*	Dynein, cytoplasmic 2, heavy chain 1	
Asphyxiating thoracic dysplasia (ATD; Jeune)	AR	208500				Unlinked to either DYNC2H1 or IFT80
Thoracolaryngopelvic dysplasia (Barnes)	AD	187760				
*See also paternal UPD14 and cerebro-costo-mandibular syndrome*						
**10. Multiple epiphyseal dysplasia and pseudoachondroplasia group**						
Pseudoachondroplasia (PSACH)	AD	177170	19p12–13.1	*COMP*	COMP	
Multiple epiphyseal dysplasia (MED) type 1 (EDM1)	AD	132400	19p13.1	*COMP*	COMP	
Multiple epiphyseal dysplasia (MED) type 2 (EDM2)	AD	600204	1p32.2–33	*COL9A2*	Collagen 9 alpha-2 chain	
Multiple epiphyseal dysplasia (MED) type 3 (EDM3)	AD	600969	20q13.3	*COL9A3*	Collagen 9 alpha-3 chain	
Multiple epiphyseal dysplasia (MED) type 5 (EDM5)	AD	607078	2p23–24	*MATN3*	Matrilin 3	
Multiple epiphyseal dysplasia (MED) type 6 (EDM6)	AD	120210	6q13	*COL9A1*	Collagen 9 alpha-1 chain	
Multiple epiphyseal dysplasia (MED), other types						Some MED-like cases unlinked to known genes
Stickler syndrome, recessive type	AR	120210	6q13	*COL9A1*	Collagen 9 alpha-1 chain	
Familial hip dysplasia (Beukes)	AD	142669	4q35			
Multiple epiphyseal dysplasia with microcephaly and nystagmus (Lowry-Wood)	AR	226960				
*See also multiple epiphyseal dysplasia, recessive type (rMED; EDM4) in sulfation disorders (group 4), familial osteochondritis dissecans in the aggrecan group, as well as ASPED in the Acromelic group*						
**11. Metaphyseal dysplasias**						
Metaphyseal dysplasia, Schmid type (MCS)	AD	156500	6q21–22.3	*COL10A1*	Collagen 10 alpha-1 chain	
Cartilage-hair hypoplasia (CHH; metaphyseal dysplasia, McKusick type)	AR	250250	9p13	*RMRP*	RNA component of RNAse H	Includes anauxetic dysplasia
Metaphyseal dysplasia, Jansen type	AD	156400	3p22–21.1	*PTHR1*	PTH/PTHrP receptor 1	Activating mutations—see also Blomstrand dysplasia (group 22, 23)
Eiken dysplasia	AR	600002	3p22–21.1	*PTHR1*	PTH/PTHrP receptor 1	Activating mutations—see also Blomstrand dysplasia (group 22, 23)
Metaphyseal dysplasia with pancreatic insufficiency and cyclic neutropenia (Shwachman–Bodian–Diamond syndrome, SBDS)	AR	260400	7q11	*SBDS*	SBDS protein	
Metaphyseal anadysplasia type 1	AD, AR	309645	11q22.2	*MMP13*	Matrix metalloproteinase 13	Includes SEMD Missouri type. Both dominant and recessive mutations described
Metaphyseal anadysplasia type 2	AR		20q13.12	*MMP9*	Matrix metalloproteinase 9	
Metaphyseal dysplasia, Spahr type	AR	250400				
Metaphyseal acroscyphodysplasia (various types)	AR	250215				
Genochondromatosis (type 1/type 2)	AD/SP	137360				
Metaphyseal chondromatosis with d-2-hydroxyglutaric aciduria	AR/SP	See 271550				
**12. Spondylometaphyseal dysplasias (SMD)**						
Spondyloenchondrodysplasia (SPENCD)	AR	271550	19p13.2	*ACP5*	Tartrate-resistant acid phosphatase (TRAP)	Includes combined immunodeficiency with autoimmunity and spondylometaphyseal dysplasia (MIM 607944)
Odontochondrodysplasia (ODCD)	AR	184260				
Spondylometaphyseal dysplasia, Sutcliffe type or corner fractures type	AD	184255				
SMD with severe genu valgum	AD	184253				Includes SMD Schmidt type and SMD Algerian type
SMD with cone-rod dystrophy	AR	608940				
SMD with retinal degeneration, axial type	AR	602271				
Dysspondyloenchondromatosis	SP					
Cheiro-spondyloenchondromatosis	SP					See also group 29
*See also SMD Kozlowski (group TRPV4) disorders in group 11 as well as SMD Sedaghatian type in group 12; there are many individual reports of SMD variants*						
**13. Spondylo-epi-(meta)-physeal dysplasias (SE(M)D)**						
Dyggve–Melchior–Clausen dysplasia (DMC)	AR	223800	18q12–21.1	*DYM*	Dymeclin	Includes Smith–McCort dysplasia
Immuno-osseous dysplasia (Schimke)	AR	242900	2q34–36	*SMARCAL1*	SWI/SNF-related regulator of chromatin subfamily A-like protein 1	
SED, Wolcott–Rallison type	AR	226980	2p12	*EIF2AK3*	Translation initiation factor 2-alpha kinase-3	
SEMD, Matrilin type	AR	608728	2p23–p24	*MATN3*	Matrilin 3	See also matrilin-related MED in group 8
SEMD, short limb—abnormal calcification type	AR	271665	1q23	*DDR2*	Discoidin domain receptor family, member 2	See also other dysplasias with stippling in group 20
SED tarda, X-linked (SED-XL)	XLR	313400	Xp22	*SEDL*	Sedlin	
Spondylo-megaepiphyseal-metaphyseal dysplasia (SMMD)	AR	613330	4p16.1	*NKX3-2*	NK3 Homeobox 2	
Spondylodysplastic Ehlers–Danlos syndrome	AR	612350	11p11.2	*SLC39A13*	Zinc transporter ZIP13	
SPONASTRIME dysplasia	AR	271510				
SEMD with joint laxity (SEMD-JL) leptodactylic or Hall type	AD	603546				
SEMD with joint laxity (SEMD-JL) Beighton type	AR	271640				
Platyspondyly (brachyolmia) with amelogenesis imperfecta	AR	601216				
Late onset SED, autosomal recessive type	AR	609223				
Brachyolmia, Hobaek, and Toledo types	AR	271530, 271630				Nosologic relationship between the Toleado and Hobaek types of brachyolmia and recessive late-onset SED are unclear, distinctive criteria lacking so far
*See also Brachyolmia (group 8), Opsismodysplasia (group 14), SEMDs (group 11), mucopolysaccharidosis type 4 (Morquio syndrome) and other conditions in group 26, as well as PPRD (SED with progressive arthropathy) in group 31*						
**14. Severe spondylodysplastic dysplasias**						
Achondrogenesis type 1A (ACG1A)	AR	200600	14q32.12	*TRIP11*	Golgi-microtubule-associated protein, 210-kDa; GMAP210	
Schneckenbecken dysplasia	AR	269250	1p31.3	*SLC35D1*	Solute carrier family 35 member D1; UDP-glucuronic acid/UDP-N-acetylgalactosamine dual transporter	
Spondylometaphyseal dysplasia, Sedaghatian type	AR	250220				
Severe spondylometaphyseal dysplasia (SMD Sedaghatian-like)	AR		7q11	*SBDS*	SBDS gene, function still unclear	
Opsismodysplasia	AR	258480				
*See also Thanatophoric dysplasia, types 1 and 2 (group 1); ACG2 and Torrance dysplasia (group 2); Fibrochondrogenesis (group 3); Achondrogenesis type 1B (ACG1B, group 4); and Metatropic dysplasia (TRPV4 group)*						
**15. Acromelic dysplasias**						
Trichorhinophalangeal dysplasia types 1/3	AD	190350	8q24	*TRPS1*	Zinc finger transcription factor	
Trichorhinophalangeal dysplasia type 2 (Langer–Giedion)	AD	150230	8q24	*TRPS1* and *EXT1*	Zinc finger transcription factor and Exostosin 1	Microdeletion syndrome; see also Multiple Cartilagineous Exostoses in group 28
Acrocapitofemoral dysplasia	AR	607778	2q33–q35	*IHH*	Indian hedgehog	
Cranioectodermal dysplasia (Levin–Sensenbrenner) type 1	AR	218330	3q21	*IFT122*	Intraflagellar transport 122 (Chlamydomonas, homolog of)	
Cranioectodermal dysplasia (Levin–Sensenbrenner) type 2	AR	613610	2p24.1	*WDR35*	WD repeat-containing protein 35	
Geleophysic dysplasia	AR	231050	9q34.2	*ADAMTSL2*	ADAMTS-like protein 2	
Geleophysic dysplasia, other types	AR					Unlinked to ADAMTSL2
Acromicric dysplasia	AD	102370				Includes acrolaryngeal dysplasia, previously known as Fantasy Island dysplasia or Tattoo dysplasia
Acrodysostosis	AD	101800				
Angel-shaped phalango-epiphyseal dysplasia (ASPED)	AD	105835				Possibly related or allelic to Brachydactyly type C
Saldino–Mainzer dysplasia	AR	266920				
*See also short rib dysplasias group*						
**16. Acromesomelic dysplasias**						
Acromesomelic dysplasia type Maroteaux (AMDM)	AR	602875	9p13–12	*NPR2*	Natriuretic peptide receptor 2	
Grebe dysplasia	AR	200700	20q11.2	*GDF5*	Growth and differentiation factor 5	Includes acromesomelic dysplasia Hunter-Thompson type; see also Brachydactylies (group 34)
Fibular hypoplasia and complex brachydactyly (Du Pan)	AR	228900	20q11.2	*GDF5*	Growth and differentiation factor 5	See also Brachydactylies (group 34)
Acromesomelic dysplasia with genital anomalies	AR	609441	4q23–24	*BMPR1B*	Bone morphogenetic protein receptor 1B	
Acromesomelic dysplasia, Osebold-Remondini type	AD	112910				
**17. Mesomelic and rhizo-mesomelic dysplasias**						
Dyschondrosteosis (Leri–Weill)	Pseudo-AD	127300	Xpter-p22.32	*SHOX*	Short stature—homeobox gene	Includes Reinhardt–Pfeiffer dysplasia, MIM 191400
Langer type (homozygous dyschondrosteosis)	Pseudo-AR	249700	Xpter-p22.32	*SHOX*	Short stature—homeobox gene	
Omodysplasia	AR	258315	13q31–q32	*GPC6*	Glypican 6	Existence of “dominant omodysplasia” (MIM 164745) remains to be confirmed
Robinow syndrome, recessive type	AR	268310	9q22	*ROR2*	Receptor tyrosine kinase-like orphan receptor 2	Includes previous costo-vertebral segmentation defect with mesomelia (COVESDEM); see also brachydactyly type B
Robinow syndrome, dominant type	AD	180700				
Mesomelic dysplasia, Korean type	AD		2q24–32		Duplication in HOXD gene cluster	
Mesomelic dysplasia, Kantaputra type	AD	156232	2q24–32		Duplications in HOXD gene cluster	
Mesomelic dysplasia, Nievergelt type	AD	163400				
Mesomelic dysplasia, Kozlowski-Reardon type	AR	249710				
Mesomelic dysplasia with acral synostoses (Verloes–David–Pfeiffer type)	AD	600383	8q13	*SULF1* and *SLCO5A1*	Heparan sulfate 6-O-endosulfatase 1 and solute carrier organic anion transporter family member 5A1	Microdeletion syndrome involving two adjacent genes
Mesomelic dysplasia, Savarirayan type (Triangular Tibia–Fibular Aplasia)	SP	605274				Possibly related to Nievergelt dysplasia. One case reported with 2q11.2 microdeletion of unclear significance
**18. Bent bones dysplasias**						
Campomelic dysplasia (CD)	AD	114290	17q24.3–25.1	*SOX9*	SRY-box 9	Includes acampomelic campomelic dysplasia (ACD) as well as mild campomelic dysplasia (MIM 602196)
Stüve–Wiedemann dysplasia	AR	601559	5p13.1	*LIFR*	Leukemia inhibitory factor receptor	Includes formerly neonatal Schwartz–Jampel syndrome or SJS type 2
Kyphomelic dysplasia, several forms		211350				Probably heterogeneous
*Bent bones at birth can be seen in a variety of conditions, including osteogenesis imperfecta, Antley–Bixler syndrome, cartilage-hair hypoplasia, Cummings syndrome, hypophosphatasia, dyssegmental dysplasia, TD, ATD, and others*						
**19. Slender bone dysplasia group**						
3-M syndrome (3M1)	AR	273750	6p21.1	*CUL7*	Cullin 7	Includes dolichospondylic dysplasia and Yakut short stature syndrome
3-M syndrome (3M2)	AR	612921	2q35	*OBSL1*	Obscurin-like 1	
Kenny–Caffey dysplasia type 1	AR	244460	1q42–q43	*TBCE*	Tubulin-specific chaperone E	
Kenny–Caffey dysplasia type 2	AD	127000				
Microcephalic osteodysplastic primordial dwarfism type 1/3 (MOPD1)	AR	210710	2q			Includes Taybi–Linder cephaloskeletal dysplasia
Microcephalic osteodysplastic primordial dwarfism type 2 (MOPD2; Majewski type)	AR	210720	21q	*PCNT2*	Pericentrin 2	
IMAGE syndrome (intrauterine growth retardation, metaphyseal dysplasia, adrenal hypoplasia, and genital anomalies)	XL/AD	300290				Possibly heterogeneous
Osteocraniostenosis	SP	602361				Occurrence in sibs reported, inheritance unclear
Hallermann–Streiff syndrome	AR	234100				Mutations in GJA1 reported in one case only
*See also Cerebro-arthro-digital dysplasia*						
**20. Dysplasias with multiple joint dislocations**						
Desbuquois dysplasia (with accessory ossification center in digit 2)	AR	251450	17q25.3	*CANT1*		
Desbuquois dysplasia with short metacarpals and elongated phalanges (Kim type)	AR	251450	17q25.3	*CANT1*		
Desbuquois dysplasia (other variants with or without accessory ossification center)	AR					Probably genetically heterogeneous
Pseudodiastrophic dysplasia	AR	264180				
*See also SED with congenital dislocations, CHST3 type (group 4); Atelosteogenesis type 3 and Larsen syndrome (group 6); SEMDs with joint laxity (group 11)*						
**21. Chondrodysplasia punctata (CDP) group**						
CDP, X-linked dominant, Conradi–Hünermann type (CDPX2)	XLD	302960	Xp11	*EBP*	Emopamil-binding protein	
CDP, X-linked recessive, brachytelephalangic type (CDPX1)	XLR	302950	Xp22.3	*ARSE*	Arylsulfatase E	
Congenital hemidysplasia, ichthyosis, limb defects (CHILD)	XLD	308050	Xp11	*NSDHL*	NAD(P)H steroid dehydrogenase-like protein	
Congenital hemidysplasia, ichthyosis, limb defects (CHILD)	XLD	308050	Xq28	*EBP*	Emopamil-binding protein	
Greenberg dysplasia	AR	215140	1q42.1	*LBR*	Lamin B receptor, 3-beta-hydroxysterol delta (14)-reductase	Includes hydrops-ectopic calcification-moth-eaten appearance dysplasia (HEM) and dappled diaphyseal dysplasia
Rhizomelic CDP type 1	AR	215100	6q22–24	*PEX7*	Peroxisomal PTS2 receptor	
Rhizomelic CDP type 2	AR	222765	1q42	*DHPAT*	Dihydroxyacetonephosphate acyltransferase (DHAPAT)	
Rhizomelic CDP type 3	AR	600121	2q31	*AGPS*	Alkylglycerone-phosphate synthase (AGPS)	
CDP tibial-metacarpal type	AD/AR	118651				Nosologic status uncertain
Astley-Kendall dysplasia	AR?					Relationship to OI and to Greenberg dysplasia unclear
*Note that stippling can occur in several syndromes such as Zellweger, Smith–Lemli–Opitz and others. See also desmosterolosis as well as SEMD short limb—abnormal calcification type in group 11*						
**22. Neonatal osteosclerotic dysplasias**						
Blomstrand dysplasia	AR	215045	3p22–21.1	*PTHR1*	PTH/PTHrP receptor 1	Caused by recessive inactivating mutations; see also Eiken dysplasia and Jansen dysplasia
Desmosterolosis	AR	602398	1p33–31.1	*DHCR24*	3-beta-hydroxysterol delta-24-reductase	See also other sterol-metabolism related conditions
Caffey disease (including infantile and attenuated forms)	AD	114000	17q21–22	*COL1A1*	Collagen 1, alpha-1 chain	See also osteogenesis imperfecta related to collagen 1 genes (group 24)
Caffey disease (severe variants with prenatal onset)	AR	114000				
Raine dysplasia (lethal and non-lethal forms)	AR	259775	7p22	*FAM20C*		Includes lethal and non-lethal cases
*See also Astley-Kendall dysplasia and CDPs in group 21*						
**23. Increased bone density group (without modification of bone shape)**						
Osteopetrosis, severe neonatal or infantile forms (OPTB1)	AR	259700	11q13	*TCIRG1*	Subunit of ATPase proton pump	
Osteopetrosis, severe neonatal or infantile forms (OPTB4)	AR	611490	16p13	*CLCN7*	Chloride channel 7	
Osteopetrosis, infantile form, with nervous system involvement (OPTB5)	AR	259720	6q21	*OSTM1*	Gray lethal/osteopetrosis associated transmembrane protein	
Osteopetrosis, intermediate form, osteoclast-poor (OPTB2)	AR	259710	13q14.11	*RANKL* (*TNFSF11*)	Receptor activator of NF-kappa-B ligand (tumor necrosis factor ligand superfamily, member 11)	
Osteopetrosis, infantile form, osteoclast-poor with immunoglobulin deficiency (OPTB7)	AR	612302	18q21.33	*RANK* (*TNFRSF11A*)	Receptor activator of NF-kappa-B	See also familial expansile osteolysis in Osteolysis group (group 28)
Osteopetrosis, intermediate form (OPTB6)	AR	611497	17q21.3	*PLEKHM1*	Pleckstrin homology domain-containing protein, family M, member 1	
Osteopetrosis, intermediate form (OPTA2)	AR	259710	16p13	*CLCN7*	Chloride channel pump	
Osteopetrosis with renal tubular acidosis (OPTB3)	AR	259730	8q22	*CA2*	Carbonic anhydrase 2	
Osteopetrosis, late-onset form type 1 (OPTA1)	AD	607634	11q13.4	*LRP5*	Low density lipoprotein receptor-related protein 5	Includes Worth type osteosclerosis (MIM 144750)
Osteopetrosis, late-onset form type 2 (OPTA2)	AD	166600	16p13	*CLCN7*	Chloride channel 7	
Osteopetrosis with ectodermal dysplasia and immune defect (OLEDAID)	XL	300301	Xq28	*IKBKG* (*NEMO*)	Inhibitor of kappa light polypeptide gene enhancer, kinase of	
Osteopetrosis, moderate form with defective leucocyte adhesion (LAD3)	AR	612840	11q12	*FERMT3* (*KIND3*)	Fermitin 3 (Kindlin 3)	
Osteopetrosis, moderate form with defective leucocyte adhesion	AR	612840	11q13	*RASGRP2* (*CalDAG-GEF1*)	Ras guanyl nucleotide-releasing protein 2	
Pyknodysostosis	AR	265800	1q21	*CTSK*	Cathepsin K	
Osteopoikilosis	AD	155950	12q14	*LEMD3*	LEM domain-containing 3	Includes Buschke–Ollendorff syndrome (MIM 166700)
Melorheostosis with osteopoikilosis	AD	155950	12q14	*LEMD3*	LEM domain-containing 3	Includes mixed sclerosing bone dysplasia
Osteopathia striata with cranial sclerosis (OSCS)	XLD	300373	Xq11.1	*WTX*	FAM123B	
Melorheostosis	SP					No germ line LEMD3 mutations identified so far
Dysosteosclerosis	AR	224300				Possibly related to “osteosclerotic metaphyseal dysplasia”
Osteomesopyknosis	AD	166450				
Osteopetrosis with infantile neuroaxonal dysplasia	AR?	600329				Same as osteopetrosis with nervous system involvement (see above)?
**24. Increased bone density group with metaphyseal and/or diaphyseal involvement**						
Craniometaphyseal dysplasia, autosomal dominant type	AD	123000	5p15.2–14.2	*ANKH*	Homolog of mouse ANK (ankylosis) gene	Gain of function mutations
Diaphyseal dysplasia Camurati-Engelmann	AD	131300	19q13	*TGFbeta1*	Transforming growth factor beta 1	
Hematodiaphyseal dysplasia Ghosal	AR	231095	7q34	*TBXAS1*	Thromboxane A synthase 1	
Hypertrophic osteoarthropathy	AR	259100	4q34–35	*HPGD*	15-alpha-hydroxyprostaglandin dehydrogenase	Includes cranio-osteoarthropathy and cases of recessive pachydermoperiostosis
Pachydermoperiostosis (hypertrophic osteoarthropathy, primary, autosomal dominant)	AD	167100				Relationship to recessive form (MIM 259100, HPGD deficiency) unclear
Oculodentoosseous dysplasia (ODOD) mild type	AD	164200	6q22–23	*GJA1*	Gap junction protein alpha-1	
Oculodentoosseous dysplasia (ODOD) severe type	AR	257850				Possibly homozygous form of mild ODOD
Osteoectasia with hyperphosphatasia (juvenile Paget disease)	AR	239000	8q24	*OPG*	Osteoprotegerin	
Sclerosteosis	AR	269500	17q12–21	*SOST*	Sclerostin	
Endosteal hyperostosis, van Buchem type	AR	239100	17q12–21	*SOST*	Sclerostin	Specific 52 kb deletion downstream of *SOST*
Trichodentoosseous dysplasia	AD	190320	17q21	*DLX3*	Distal-less homeobox 3	
Craniometaphyseal dysplasia, autosomal recessive type	AR	218400	6q21–22			
Diaphyseal medullary stenosis with bone malignancy	AD	112250	9p21–p22			
Craniodiaphyseal dysplasia	AD	122860				
Craniometadiaphyseal dysplasia, Wormian bone type	AR	—				
Endosteal sclerosis with cerebellar hypoplasia	AR	213002				
Lenz–Majewski hyperostotic dysplasia	SP	151050				
Metaphyseal dysplasia, Braun–Tinschert type	XL	605946				
Pyle disease	AR	265900				
**25. Osteogenesis imperfecta and decreased bone density group**						
*For comments the classification of osteogenesis imperfecta, please refer to the text*						
Osteogenesis imperfecta, non-deforming form (OI type 1)	AD			*COL1A1*, *COL1A2*	COL1A1: collagen 1 alpha-1 chain, COL1A2: collagen 1 alpha-2 chain, CRTAP: cartilage-associated protein, LEPRE1: leucine proline-enriched proteoglycan (leprecan) 1, PPIB: peptidylprolyl isomerase B (cyclophilin B), FKBP10: FK506 binding protein 10, SERPINH: serpin peptidase inhibitor clade H 1, SP7: SP7 transcription factor (Osterix)	
Osteogenesis imperfecta, perinatal lethal form (OI type 2)	AD, AR			*COL1A1*, *COL1A2*, *CRTAP*, *LEPRE1*, *PPIB*		
Osteogenesis imperfecta, progressively deforming type (OI type 3)	AD, AR			*COL1A1*, *COL1A2*, *CRTAP*, *LEPRE1*, *PPIB*, *FKBP10*, *SERPINH1*		See also Bruck syndrome type 1 (below)
Osteogenesis imperfecta, moderate form (OI type 4)	AD, AR			*COL1A1*, *COL1A2*, *CRTAP*, *FKBP10*, *SP7*		
Osteogenesis imperfecta with calcification of the interosseous membranes and/or hypertrophic callus (OI type 5)	AD	610967				
Osteogenesis imperfecta, other types						
Bruck syndrome type 1 (BS1)	AR	259450	17q12	*FKBP10*	FK506 binding protein 10	See autosomal recessive OI, above; intrafamilial variability between OI3 and BS1 documented
Bruck syndrome type 2 (BS2)	AR	609220	3q23–24	*PLOD2*	Procollagen lysyl hydroxylase 2	
Osteoporosis-pseudoglioma syndrome	AR	259770	11q12–13	*LRP5*	LDL-receptor related protein 5	
Calvarial doughnut lesions with bone fragility	AD	126550				
Idiopathic juvenile osteoporosis	SP	259750				Some patients reported with heterozygous mutations in the *LRP5* gene
Cole-Carpenter dysplasia (bone fragility with craniosynostosis)	SP	112240				See also craniosynostosis syndromes in group 30
Spondylo-ocular dysplasia	AR	605822				Unlinked to collagen 1 and collagen 2 genes or *LRP5*
Osteopenia with radiolucent lesions of the mandible	AD	166260				
Ehlers–Danlos syndrome, progeroid form	AR	130070	5q35	*B4GALT7*	Xylosylprotein 4-beta-galactosyltransferase deficiency	
Geroderma osteodysplasticum	AR	231070	1q24.2	*GORAB*	SCYL1-binding protein 1	
Cutis laxa, autosomal recessive form, type 2B (ARCL2B)	AR	612940	17q25.3	*PYCR1*	Pyrroline-5-carboxylate reductase 1	Skeletal features overlapping with progeroid EDS and geroderma osteodysplasticum
Cutis laxa, autosomal recessive form, type 2A (ARCL2A) (Wrinkly skin syndrome)	AR	278250, 219200	12q24.3	*ATP6VOA2*	ATPase, H+ transporting, lysosomal, V0 subunit A2	Skeletal features overlapping with progeroid EDS and geroderma osteodysplasticum
Singleton–Merten dysplasia	AD	182250				
**26. Abnormal mineralization group**						
Hypophosphatasia, perinatal lethal and infantile forms	AR	241500	1p36.1–p34	*ALPL*	Alkaline phosphatase, tissue non-specific (TNSALP)	Intrafamilial variability
Hypophosphatasia, adult form	AD	146300	1p36.1–p34	*ALPL*	Alkaline phosphatase, tissue non-specific (TNSALP)	Includes odontohypophosphatasia
Hypophosphatemic rickets, X-linked dominant	XLD	307800	Xp22	*PHEX*	X-linked hypophosphatemia membrane protease	
Hypophosphatemic rickets, autosomal dominant	AD	193100	12p13.3	*FGF23*	Fibroblast growth factor 23	
Hypophosphatemic rickets, autosomal recessive, type 1 (ARHR1)	AR	241520	4q21	*DMP1*	Dentin matrix acidic phosphoprotein 1	
Hypophosphatemic rickets, autosomal recessive, type 2 (ARHR2)	AR	613312	6q23	*ENPP1*	Ectonucleotide pyrophosphatase/phosphodiesterase 1	
Hypophosphatemic rickets with hypercalciuria, X-linked recessive	XLR	300554	Xp11.22	*ClCN5*	Chloride channel 5	Part of Dent's disease complex
Hypophosphatemic rickets with hypercalciuria, autosomal recessive (HHRH)	AR	241539	9q34	*SLC34A3*	Sodium-phosphate cotransporter	
Neonatal hyperparathyroidism, severe form	AR	239200	3q13.3–21	*CASR*	Calcium-sensing receptor	
Familial hypocalciuric hypercalcemia with transient neonatal hyperparathyroidism	AD	145980	3q13.3–21	*CASR*	Calcium-sensing receptor	
Calcium pyrophosphate deposition disease (familial chondrocalcinosis) type 2	AD	118600	5p15.2–14.2	*ANKH*	Homolog of mouse ANK (ankylosis) gene	Loss of function mutations (see craniometaphyseal dysplasia in group 24)
*See also Jansen dysplasia and Eiken dysplasia*						
**27. Lysosomal storage diseases with skeletal involvement (dysostosis multiplex group)**						
Mucopolysaccharidosis type 1H/1S	AR	607014	4p16.3	*IDA*	Alpha-1-Iduronidase	
Mucopolysaccharidosis type 2	XLR	309900	Xq27.3–28	*IDS*	Iduronate-2-sulfatase	
Mucopolysaccharidosis type 3A	AR	252900	17q25.3	*HSS*	Heparan sulfate sulfatase	
Mucopolysaccharidosis type 3B	AR	252920	17q21	*NAGLU*	N-Ac-beta-d-glucosaminidase	
Mucopolysaccharidosis type 3C	AR	252930	8p11–q13	*HSGNAT*	Ac-CoA: alpha-glucosaminide N-acetyltransferase	
Mucopolysaccharidosis type 3D	AR	252940	12q14	*GNS*	N-Acetylglucosamine 6-sulfatase	
Mucopolysaccharidosis type 4A	AR	253000	16q24.3	*GALNS*	Galactosamine-6-sulfate sulfatase	
Mucopolysaccharidosis type 4B	AR	253010	3p21.33	*GLBI*	beta-Galactosidase	
Mucopolysaccharidosis type 6	AR	253200	5q13.3	*ARSB*	Arylsulfatase B	
Mucopolysaccharidosis type 7	AR	253220	7q21.11	*GUSB*	beta-Glucuronidase	
Fucosidosis	AR	230000	1p34	*FUCA*	alpha-Fucosidase	
alpha-Mannosidosis	AR	248500	19p13.2–12	*MANA*	alpha-Mannosidase	
beta-Mannosidosis	AR	248510	4q22–25	*MANB*	beta-Mannosidase	
Aspartylglucosaminuria	AR	208400	4q23–27	*AGA*	Aspartyl-glucosaminidase	
GMI Gangliosidosis, several forms	AR	230500	3p21–14.2	*GLB1*	beta-Galactosidase	
Sialidosis, several forms	AR	256550	6p21.3	*NEU1*	Neuraminidase (sialidase)	
Sialic acid storage disease (SIASD)	AR	269920	6q14–q15	*SLC17A5*	Sialin (sialic acid transporter)	
Galactosialidosis, several forms	AR	256540	20q13.1	*PPGB*	beta-Galactosidase protective protein	
Multiple sulfatase deficiency	AR	272200	3p26	*SUMF1*	Sulfatase-modifying factor-1	
Mucolipidosis II (I-cell disease), alpha/beta type	AR	252500	4q21–23	*GNPTAB*	N-Acetylglucosamine 1-phosphotransferase, alpha/beta subunits	
Mucolipidosis III (Pseudo-Hurler polydystrophy), alpha/beta type	AR	252600	4q21–23	*GNPTAB*	N-Acetylglucosamine 1-phosphotransferase, alpha/beta subunits	
Mucolipidosis III (Pseudo-Hurler polydystrophy), gamma type	AR	252605	4q21–23	*GNPTG*	N-Acetylglucosamine 1-phosphotransferase, gamma subunit	
**28. Osteolysis group**						
Familial expansile osteolysis	AD	174810	18q22.1	*RANK* (*TNFRSF11A*)		Includes expansile skeletal hyperphosphatasia (MIM 602080)
Mandibuloacral dysplasia type A	AD	248370	1q21.2	*LMNA*	Lamin A/C	
Mandibuloacral dysplasia type B	AR	608612	1p34	*ZMPSTE24*	Zinc metalloproteinase	
Progeria, Hutchinson–Gilford type	AD	176670	1q21.2	*LMNA*	Lamin A/C	
Torg–Winchester syndrome	AR	259600	16q13	*MMP2*	Matrix metalloproteinase 2	Includes Nodulosis–Arthropathy–Osteolysis syndrome (MIM 605156)
Hajdu–Cheney syndrome	AD	102500				
Multicentric carpal-tarsal osteolysis with and without nephropathy	AD	166300				
Lipomembraneous osteodystrophy with leukoencephalopathy (presenile dementia with bone cysts; Nasu–Hakola)	AR	221770	6p21.2	*TREM2*	Triggering receptor expressed on myeloid cells 2	
Lipomembraneous osteodystrophy with leukoencephalopathy (presenile dementia with bone cysts; Nasu–Hakola)	AR	221770	19q13.1	*TYROBP*	Tyro protein tyrosine kinase-binding protein	
*See also Pycnodysostosis, cleidocranial dysplasia, and Singleton–Merten syndrome. Note: several neurologic conditions may cause acroosteolysis*						
**29. Disorganized development of skeletal components group**						
Multiple cartilaginous exostoses 1	AD	133700	8q23–24.1	*EXT1*	Exostosin-1	
Multiple cartilaginous exostoses 2	AD	133701	11p12–11	*EXT2*	Exostosin-2	
Multiple cartilaginous exostoses 3	AD	600209	19p			
Cherubism	AD	118400	4p16	*SH3BP2*	SH3 domain-binding protein 2	
Fibrous dysplasia, polyostotic form	SP	174800	20q13	*GNAS1*	Guanine nucleotide-binding protein, alpha-stimulating activity subunit 1	Somatic mosaicism and imprinting phenomena; includes McCune–Albright syndrome
Progressive osseous heteroplasia	AD	166350	20q13	*GNAS1*	Guanine nucleotide-binding protein, alpha-stimulating activity subunit 1	Gene subject to imprinting
Gnathodiaphyseal dysplasia	AD	166260	11p15.1–14.3	*TMEM16E*	Transmembrane protein 16E	
Metachondromatosis	AD	156250	12q24	*PTPN11*	Protein-tyrosine phosphatase nonreceptor-type 11	
Osteoglophonic dysplasia	AD	166250	8p11	*FGFR1*	Fibroblast growth factor receptor 1	See also Craniosynostosis syndromes in group 30
Fibrodysplasia ossificans progressiva (FOP)	AD, SP	135100	2q23–24	*ACVR1*	Activin A (BMP type 1) receptor	
Neurofibromatosis type 1 (NF1)	AD	162200	17q11.2	*NF1*	Neurofibromin	
Carpotarsal osteochondromatosis	AD	127820				
Cherubism with gingival fibromatosis (Ramon syndrome)	AR	266270				
Dysplasia epiphysealis hemimelica (Trevor)	SP	127800				
Enchondromatosis (Ollier)	SP	166000				*PTHR1* and *PTPN11* mutations found in a few cases only, role still unclear
Enchondromatosis with hemangiomata (Maffucci)	SP	166000				*PTPN11* mutations found in a few cases only, role unclear
*See also Proteus syndrome in group 30*						
**30. Overgrowth syndromes with skeletal involvement**						
Weaver syndrome	SP/AD	277590				Some cases reported with *NSD1* mutations (see Sotos syndrome)
Sotos syndrome	AD	117550	5q35	*NSD1*	Nuclear receptor-binding su-var, enhancer of zeste, and trithorax domain protein 1	Some cases may have *NFIX* mutations (see Marshall–Smith syndrome)
Marshall–Smith syndrome	SP	602535	19p13.3	*NFIX*	Nuclear factor I/X	Some clinical overlap with Sotos syndrome (see above)
Proteus syndrome	SP	176920				Some Proteus-like cases have mutations in the *PTEN* gene
Marfan syndrome	AD	154700	15q21.1	*FBN1*	Fibrillin 1	
Congenital contractural arachnodactyly	AD	121050	5q23.3	*FBN2*	Fibrillin 2	
Loeys–Dietz syndrome types 1A and 2A	AD	609192, 610168,	9q22	*TGFBR1*	TGFbeta receptor subunit 1	
Loeys–Dietz syndrome types 1B and 2B	AD	608967, 610380	3p22	*TGFBR2*	TGFbeta receptor subunit 2	
Overgrowth syndrome with 2q37 translocations	SP	—	2q37	*NPPC*	Natriuretic peptide precursor C	Overgrowth probably caused by overexpression of *NPPC*
Overgrowth syndrome with skeletal dysplasia (Nishimura–Schmidt, endochondral gigantism)	SP?					Nosologic status unclear but conspicuous skeletal phenotype(s)
*See also Shprintzen–Goldberg syndrome in Craniosynostosis group*						
**31. Genetic inflammatory/rheumatoid-like osteoarthropathies**						
Progressive pseudorheumatoid dysplasia (PPRD; SED with progressive arthropathy)	AR	208230	6q22–23	*WISP3*	WNT1-inducible signaling pathway protein 3	
Chronic infantile neurologic cutaneous articular syndrome (CINCA)/neonatal onset multisystem inflammatory disease (NOMID)	AD	607115	1q44	*CIAS1*	Cryopyrin	
Sterile multifocal osteomyelitis, periostitis, and pustulosis (CINCA/NOMID-like)	AR	147679	2q14.2	*IL1RN*	Interleukin 1 receptor antagonist	
Chronic recurrent multifocal osteomyelitis with congenital dyserythropoietic anemia (CRMO with CDA; Majeed syndrome)	AR	609628	18p11.3	*LPIN2*	Lipin 2	
Hyperostosis/hyperphosphatemia syndrome	AR	610233	2q24–q31	*GALNT3*	UDP-N-acetyl-alpha-d-galactosamine:polypeptide N-acetylgalactosaminyltransferase 3	
Infantile systemic hyalinosis/Juvenile hyaline fibromatosis (ISH/JHF)	AR	236490	4q21	*ANTXR2*	Anthrax toxin receptor 2	Includes Juvenile hyaline fibromatosis (JHF, 228600) and Puretic syndrome
**32. Cleidocranial dysplasia and isolated cranial ossification defects group**						
Cleidocranial dysplasia	AD	119600	6p21	*RUNX2*	Runt related transcription factor 2	
CDAGS syndrome (craniosynostosis, delayed fontanel closure, parietal foramina, imperforate anus, genital anomalies, skin eruption)	AR	603116	22q12–q13			
Yunis–Varon dysplasia	AR	216340				
Parietal foramina (isolated)	AD	168500	11q11.2	*ALX4*	Aristaless-like 4	See also Frontonasal dysplasia type 1 (group 34)
Parietal foramina (isolated)	AD	168500	5q34–35	*MSX2*	Muscle segment homeobox 2	
*See also pycnodysostosis, wrinkly skin syndrome, and several others*						
**33. Craniosynostosis syndromes**						
Pfeiffer syndrome (FGFR1-related)	AD	101600	8p12	*FGFR1*	Fibroblast growth factor receptor 1	Most have *FGFR1* P252R mutation (phenotype generally milder than FGFR2-related Pfeiffer)
Pfeiffer syndrome (FGFR2-related)	AD	101600	10q26.12	*FGFR2*	Fibroblast growth factor receptor 2	Includes Jackson–Weiss syndrome (MIM 123150) and Antley–Bixler variants caused by *FGFR2* mutations (see below)
Apert syndrome	AD	101200	10q26.12	*FGFR2*	Fibroblast growth factor receptor 2	
Craniosynostosis with cutis gyrata (Beare–Stevenson)	AD	123790	10q26.12	*FGFR2*	Fibroblast growth factor receptor 2	
Crouzon syndrome	AD	123500	10q26.12	*FGFR2*	Fibroblast growth factor receptor 2	
Crouzon-like craniosynostosis with acanthosis nigricans (Crouzonodermoskeletal syndrome)	AD	612247	4p16.3	*FGFR3*	Fibroblast growth factor receptor 3	Defined by specific *FGFR3* A391E mutation
Craniosynostosis, Muenke type	AD	602849	4p16.3	*FGFR3*	Fibroblast growth factor receptor 3	Defined by specific *FGFR3* P250R mutation
Antley–Bixler syndrome	AR	201750	7q11.23	*POR*	Cytochrome P450 oxidoreductase	Similar cases with F*GFR2* mutations classified as Pfeiffer syndrome (MIM 207410)
Craniosynostosis Boston type	AD	604757	5q35.2	*MSX2*	MSX2	Heterozygous P148H mutation in a single family
Saethre–Chotzen syndrome	AD	101400	7p21.1	*TWIST1*	TWIST	
Shprintzen–Goldberg syndrome	AD	182212				Some cases reported with *FBN1* mutations
Baller–Gerold syndrome	AR	218600	8q24.3	*RECQL4*	RECQ protein-like 4	*RECQL4* might not account for all cases of Baller–Gerold
Carpenter syndrome	AR	201000		*RAB23*		
*See also Cole–Carpenter syndrome in group 24, CDAGS syndrome in group 29, and Craniofrontonasal syndrome in group 34*						
**34. Dysostoses with predominant craniofacial involvement**						
Mandibulo-facial dysostosis (Treacher Collins, Franceschetti-Klein)	AD	154500	5q32	*TCOF1*	Treacher Collins-Franceschetti syndrome 1	
Mandibulo-facial dysostosis (Treacher-Collins, Franceschetti-Klein)	AD	154500	13q12.2	*POLR1D*	Polymerase (RNA) I polypeptide D	
Mandibulo-facial dysostosis (Treacher-Collins, Franceschetti-Klein)	AR	154500	6p21.1	*POLR1C*	Polymerase (RNA) I polypeptide C	
Oral-facial-digital syndrome type I (OFD1)	XLR	311200	Xp22.3	*CXORF5*	chr. X open reading frame 5	
Weyer acrofacial (acrodental) dysostosis	AD	193530	4p16	*EVC1*	Ellis–van Creveld 1 protein	
Endocrine-cerebro-osteodysplasia (ECO)	AR	612651	6p12.3	*ICK*	Intestinal cell kinase	
Craniofrontonasal syndrome	XLD	304110	Xq13.1	*EFNB1*	Ephrin B1	
Frontonasal dysplasia, type 1	AR	136760	1p13.3	*ALX3*	Aristaless-like-3	
Frontonasal dysplasia, type 2	AR	613451	11p11.2	*ALX4*	Aristaless-like-4	
Frontonasal dysplasia, type 3	AR	613456	12q21.3	*ALX1*	Aristaless-like 1	
Hemifacial microsomia	SP/AD	164210				Includes Goldenhar syndrome and Oculo-Auriculo-Vertebral spectrum; probably genetically heterogeneous
Miller syndrome (postaxial acrofacial dysostosis)	AR	263750	16q22	*DHODH*	Dihydroorotate dehydrogenase	
Acrofacial dysostosis, Nager type	AD/AR	154400				
Acrofacial dysostosis, Rodriguez type	AR	201170				
*See also Oral-facial-digital syndrome type IV in the Short Rib Dysplasias group*						
**35. Dysostoses with predominant vertebral with and without costal involvement**						
Currarino triad	AD	176450	7q36	*HLXB9*	Homeobox gene HB9	
Spondylocostal dysostosis type 1 (SCD1)	AR	277300	19q13	*DLL3*	Delta-like 3	
Spondylocostal dysostosis type 2 (SCD2)	AR	608681	15q26	*MESP2*	Mesoderm posterior (expressed in) 2	
Spondylocostal dysostosis type 3 (SCD3)	AR?	609813	7p22	*LFNG*	Lunatic fringe	
Spondylocostal dysostosis type 4 (SCD4)	AR		17p13.1	*HES7*	Hairy-and-enhancer-of-split-7	
Spondylothoracic dysostosis	AR		15q26	*MESP2*	Mesoderm posterior (expressed in) 2	
Klippel–Feil anomaly with laryngeal malformation	AD	148900	8q22.1	*GDF6*	Growth and differentiation factor 6	Role of *GDF6* mutations in dominant spondylothoracic dysostosis unclear
Spondylocostal/thoracic dysostosis, other forms	AD/AR					See also *GDF6*, above
Cerebro-costo-mandibular syndrome (rib gap syndrome)	AD/AR	117650				
Cerebro-costo-mandibular-like syndrome with vertebral defects	AR	611209	17q25	*COG1*	Component of oligomeric Golgi complex 1	Also classified as CDG type IIg
Diaphanospondylodysostosis	AR	608022	7p14	*BMPER*	Bone morphogenetic protein-binding endothelial cell precursor-derived regulator	Possibly overlaps with ischiospinal dysostosis
*See also Spondylocarpotarsal dysplasia in group 7 and spondylo-metaphyseal-megaepiphyseal dysplasia in group 13*						
**36. Patellar dysostoses**						
Ischiopatellar dysplasia (small patella syndrome)	AD	147891	17q21–q22	*TBX4*	T-box gene 4	
Small patella—like syndrome with clubfoot	AD		5q31	*PITX1*	Paired-like homeodomain transcription factor 1 (pituitary homeobox 1)	Includes isolated dominant familial clubfoot
Nail-patella syndrome	AD	161200	9q34.1	*LMX1B*	LIM homeobox transcription factor 1	
Genitopatellar syndrome	AR?	606170				
Ear-patella-short stature syndrome (Meier-Gorlin)	AR	224690				
*See also MED group for conditions with patellar changes as well as ischio-pubic-patellar dysplasia as mild expression of campomelic dysplasia*						
**37. Brachydactylies (with or without extraskeletal manifestations)**						
Brachydactyly type A1	AD	112500	2q35–36	*IHH*	Indian Hedgehog	
Brachydactyly type A1	AD		5p31			
Brachydactyly type A2	AD	112600	4q23	*BMPR1B*	Bone morphogenetic protein receptor, 1B	
Brachydactyly type A2	AD	112600		*BMP2*	Bone morphogenetic protein type 2	
Brachydactyly type A2	AD	112600	20q11.2	*GDF5*	Growth and differentiation factor 5	
Brachydactyly type A3	AD	112700				
Brachydactyly type B	AD	113000	9q22	*ROR2*	Receptor tyrosine kinase-like orphan receptor 2	See also Robinow syndrome/COVESDEM
Brachydactyly type B2	AD	611377	17q	*NOG*	Noggin	
Brachydactyly type C	AD, AR	113100	20q11.2	*GDF5*	Growth and differentiation factor 5	See also ASPED (group 14) and other *GDF5* disorders
Brachydactyly type D	AD	113200	2q31	*HOXD13*	Homeobox D13	
Brachydactyly type E	AD	113300	12p11.22	*PTHLH*	Parathyroid hormone-like hormone (parathyroid hormone related peptide, PTHRP)	
Brachydactyly type E	AD	113300	2q31	*HOXD13*	Homeobox D13	
Brachydactyly—mental retardation syndrome	AD	600430	2q37.3	*HDAC4*	Histone deacetylase 4	Some patients have microdeletions involving contiguous genes (chr. 2q37 deletion syndrome)
Hyperphosphatasia with mental retardation, brachytelephalangy, and distinct face	AR		1p36.11	*PIGV*	Phosphatidylinositol-glycan biosynthesis class V protein (GPI mannosyltransferase 2)	
Brachydactyly-hypertension syndrome (Bilginturian)	AD	112410	12p12.2–11.2			Possibly *PTHLH*
Brachydactyly with anonychia (Cooks syndrome)	AD	106995	17q24.3	*SOX9*		Regulatory mutations
Microcephaly-oculo-digito-esophageal-duodenal syndrome (Feingold syndrome)	AD	164280	2p24.1	*MYCN*	nMYC oncogene	
Hand-foot-genital syndrome	AD	140000	7p14.2	*HOXA13*	Homeobox A13	
Brachydactyly with elbow dysplasia (Liebenberg syndrome)	AD	186550				
Keutel syndrome	AR	245150	12p13.1–12.3	*MGP*	Matrix Gla protein	
Albright hereditary osteodystrophy (AHO)	AD	103580	20q13	*GNAS1*	Guanine nucleotide binding protein of adenylate cyclase—subunit	See also polyostotic fibrous dysplasia and progressive osseous heteroplasia, group 28
Rubinstein–Taybi syndrome	AD	180849	16p13.3	*CREBBP*	CREB-binding protein	
Rubinstein–Taybi syndrome	AD	180849	22q13	*EP300*	E1A-binding protein, 300-kDa	
Catel–Manzke syndrome	XLR?	302380				
Brachydactyly, Temtamy type	AR	605282				
Christian type brachydactyly	AD	112450				
Coffin–Siris syndrome	AR	135900				
Mononen type brachydactyly	XLD?	301940				
Poland anomaly	SP	173800				
*See also group 20 for other conditions with brachydactyly as well as brachytelephalangic CDP*						
**38. Limb hypoplasia—reduction defects group**						
Ulnar-mammary syndrome	AD	181450		*TBX3*	T-box gene 3	
de Lange syndrome	AD	122470	5p13.1	*NIPBL*	Nipped-B-like	
Fanconi anemia (see note below)	AR	227650	Several	Several		Several complementation groups and genes
Thrombocytopenia-absent radius (TAR)	AR?/AD?	274000	1q21.1	Several		Microdeletion on 1q21.1
Thrombocythemia with distal limb defects	AD		3q27	*THPO*	Thrombopoietin	Distal limb defects postulated as consequence of vascular occlusions
Holt–Oram syndrome	AD	142900	12q24.1	*TBX5*	T-box gene 5	
Okihiro syndrome (Duane—radial ray anomaly)	AD	607323	20q13	*SALL4*	SAL-like 4	
Cousin syndrome	AR	260660	1p13	*TBX15*	T-box gene 15	
Roberts syndrome	AR	268300	8p21.1	*ESCO2*	Homolog of establishment of cohesion—2	
Split-hand-foot malformation with long bone deficiency (SHFLD1)	AD	119100	1q42.2–q43			
Split-hand-foot malformation with long bone deficiency (SHFLD2)	AD	610685	6q14.1			
Split-hand-foot malformation with long bone deficiency (SHFLD3)	AD	612576	17p13.1			
Tibial hemimelia	AR	275220				
Tibial hemimelia-polysyndactyly-triphalangeal thumb	AD	188770				
Acheiropodia	AR	200500	7q36	*LMBR1*	Putative receptor protein	Partial LMBR1 deletion affecting expression of Sonic Hedgehog (SHH) gene
Tetra-amelia	XL	301090				
Tetra-amelia	AR	273395	17q21	*WNT3*	Wingless-type MMTV integration site family, member 3	
Ankyloblepharon-ectodermal dysplasia-cleft lip/palate (AEC)	AD	106260	3q27	*P63* (*TP63*)	Tumor protein p63	
Ectrodactyly-ectodermal dysplasia cleft-palate syndrome Type 3 (EEC3)	AD	604292	3q27	*P63* (*TP63*)	Tumor protein p63	
Ectrodactyly-ectodermal dysplasia cleft-palate syndrome type 1 (EEC1)	AD	129900	7q11.2–12.3			
Ectrodactyly-ectodermal dysplasia-macular dystrophy syndrome (EEM)	AR	225280	16q22	*CDH3*	Cadherin 3	
Limb-mammary syndrome (including ADULT syndrome)	AD	603273	3q27	*P63* (*TP63*)	Tumor protein p63	
Split hand-foot malformation, isolated form, type 4 (SHFM4)	AD	605289	3q27	*P63* (*TP63*)	Tumor protein p63	
Split hand-foot malformation, isolated form, type 1 (SHFM1)	AD	183600	7q21.3–22.1			
Split hand-foot Malformation, isolated form, type 2 (SHFM2)	XL	313350	Xq26			
Split hand-foot malformation, isolated form, type 3 (SHFM3)	AD	600095	10q24	*FBXW4*	Dactylin	
Split hand-foot malformation, isolated form, type 5 (SHFM5)	AD	606708	2q31			
Al-Awadi Raas–Rothschild limb-pelvis hypoplasia–aplasia	AR	276820	3p25	*WNT7A*	Wingless-type MMTV integration site family, member 7A	
Fuhrmann syndrome	AR	228930	3p25	*WNT7A*	Wingless-type MMTV integration site family, member 7A	
RAPADILINO syndrome	AR	266280	8q24.3	*RECQL4*	RECQ protein-like 4	
Adams–Oliver syndrome	AD/AR	100300				
Femoral hypoplasia-unusual face syndrome (FHUFS)	SP/AD?	134780				Some phenotypic overlap with FFU syndrome (below)
Femur-fibula-ulna syndrome (FFU)	SP?	228200				
Hanhart syndrome (hypoglossia–hypodactylia)	AD	103300				
Scapulo-iliac dysplasia (Kosenow)	AD	169550				
*Note: the particularly complex genetic basis of Fanconi anemia and its complementation groups are acknowledged but not further listed in this Nosology. The Reader is referred to MIM or to specialized reviews. See also CHILD in group 20 and the mesomelic and acromesomelic dysplasias*						
**39. Polydactyly–Syndactyly–Triphalangism group**						
Preaxial polydactyly type 1 (PPD1)	AD	174400	7q36	*SHH*	Sonic Hedgehog	Regulatory mutation
Preaxial polydactyly type 1 (PPD1)	AD	174400				Some instances not linked to SHH
Preaxial polydactyly type 2 (PPD2)/triphalangeal thumb (TPT)	AD	174500	7q36	*SHH*	Sonic Hedgehog	Regulatory mutation
Preaxial polydactyly type 3 (PPD3)	AD	174600				
Preaxial polydactyly type 4 (PPD4)	AD	174700	7p13	*GLI3*	Gli-Kruppel family member 3	
Greig cephalopolysyndactyly syndrome	AD	175700	7p13	*GLI3*	Gli-Kruppel family member 3	
Pallister–Hall syndrome	AD	146510	7p13	*GLI3*	Gli-Kruppel family member 3	
Synpolydactyly (complex, fibulin1—associated)	AD	608180	22q13.3	*FBLN1*	Fibulin 1	
Synpolydactyly	AD	186000	2q31	*HOXD13*	Homeobox D13	
Townes–Brocks syndrome (Renal-Ear-Anal-Radial syndrome)	AD	107480	16q12.1	*SALL1*	SAL-like 1	
Lacrimo-auriculo-dento-digital syndrome (LADD)	AD	149730	10q26.12	*FGFR2*	Fibroblast growth factor receptor 2	
Lacrimo-auriculo-dento-digital syndrome (LADD)	AD	149730	4p16.3	*FGFR3*	Fibroblast growth factor receptor 3	
Lacrimo-auriculo-dento-digital syndrome (LADD)	AD	149730	5p13–p12	*FGF10*	Fibroblast growth factor 10	
Acrocallosal syndrome	AR	200990	7p13			
Acro-pectoral syndrome	AD	605967	7q36			
Acro-pectoro-vertebral dysplasia (F-syndrome)	AD	102510	2q36			
Mirror-image polydactyly of hands and feet (Laurin–Sandrow syndrome)	AD	135750	7q36	*SHH*	Sonic Hedgehog	
Mirror-image polydactyly of hands and feet (Laurin–Sandrow syndrome)						Unlinked to SHH
Cenani–Lenz syndactyly	AR	212780	11p11.2	*LRP4*	Low density lipoprotein receptor-related protein 4	
Cenani–Lenz like syndactyly	SP (AD?)		15q13–q14	*GREM1, FMN1*	Gremlin 1, Formin 1	Monoallelic duplication of both loci (observed in one case only so far)
Oligosyndactyly, radio-ulnar synostosis, hearing loss, and renal defects syndrome	SP (AR?)		15q13–q14	*FMN1*	Formin 1	Deletion
Syndactyly, Malik–Percin type	AD	609432	17p13.3			
STAR syndrome (syndactyly of toes, telecanthus, ano-, and renal malformations)	XL	300707	Xq28	*FAM58A*		
Syndactyly type 1 (III–IV)	AD	185900	2q34–36			
Syndactyly type 3 (IV–V)	AD	185900	6q21–23	*GJA1*		
Syndactyly type 4 (I–V) Haas type	AD	186200	7q36	*SHH*	Sonic Hedgehog	
Syndactyly type 5 (syndactyly with metacarpal and metatarsal fusion)	AD	186300	2q31	*HOXD13*		
Syndactyly with craniosynostosis (Philadelphia type)	AD	601222	2q35–36.3			
Syndactyly with microcephaly and mental retardation (Filippi syndrome)	AR	272440				
Meckel syndrome type 1	AR	249000	17q23	*MKS1*		
Meckel syndrome type 2	AR	603194	11q			
Meckel syndrome type 3	AR	607361	8q21	*TMEM67*		
Meckel syndrome type 4	AR	611134	12q	*CEP290*		
Meckel syndrome type 5	AR	611561	16q12.1	*RPGRIP1L*		
Meckel syndrome type 6	AR	612284	4p15	*CC2D2A*		
*Note: the Smith–Lemli–Opitz syndrome can present with polydactyly and/or syndactyly. See also the SRPS group*						
**40. Defects in joint formation and synostoses**						
Multiple synostoses syndrome type 1	AD	186500	17q22	*NOG*	Noggin	
Multiple synostoses syndrome type 2	AD	186500	20q11.2	*GDF5*	Growth and differentiation factor 5	
Multiple synostoses syndrome type 3	AD	612961	13q11–q12	*FGF9*		
Proximal symphalangism type 1	AD	185800	17q22	*NOG*	Noggin	
Proximal symphalangism type 2	AD	185800	20q11.2	*GDF5*	Growth and differentiation factor 5	
Radio-ulnar synostosis with amegakaryocytic thrombocytopenia	AD	605432	7p15–14.2	*HOXA11*	Homeobox A11	
*See also Spondylo-Carpal-Tarsal dysplasia; mesomelic dysplasia with acral synostoses; and others*						

The organization of groups has been further changed in comparison to the 2006 version. Two new groups based on a common affected molecule or biochemical pathway have been created (*TRPV4 group* and *Aggrecan group*). The TRPV4 group includes disorders that are relatively common and that constitute a new prototypic spectrum ranging from mild to lethal. Aggrecan is one of the important structural molecules in cartilage and it would not be surprising if more disorders would find their way into this group in the future. Thus, groups 1–8 are based on a common underlying gene or pathway.

Groups 9–17 are based on the localization of radiographic changes to specific bone structures (vertebrae, epiphyses, metaphyses, diaphysis, or combination thereof) or of the involved segment (rhizo, meso, or acro). Groups 18–20 are defined by macroscopic criteria in combination with clinical features (bent bones, slender bones, presence of multiple dislocations). Groups 21–25 and 28 take into account features of mineralization (increased or reduced bone density, impaired mineralization, stippling, osteolysis). Group 27 encompasses the large group of lysosomal disorders with skeletal involvement. Group 29 comprises disorders with so-called abnormal (previously “anarchic”) development of skeletal components such as exostoses, ecnhondromas, and ectopic calcification. It is particularly heterogeneous and may need to be revised in the future with the help of newer molecular data.

Group 23, comprising the osteopetrosis (OP) variants and related disorders, has been expanded following the identification of distinct genetic defects in various variants of osteopetrosis. The diversity of molecular mechanisms involved and the presence of clinical, biochemical and/or histologic features that distinguish between the various OP forms justify the subdivision of the “OP phenotype” in the many subtypes.

Group 25 (Osteogenesis Imperfecta and decreased bone density group) has had special attention. The Sillence classification, published 30 years ago, provided a first systematic clinical classification and made correlations to the inheritance pattern of individual clinical types [Sillence and Rimoin, 1978; Sillence et al., 1979a,b]. Today, a surprising genetic complexity of the molecular bases of OI has been revealed, and at the same time the extensive phenotypic variation arising from single loci has been documented clearly. It seemed therefore untenable to try and maintain tight correlations between “Sillence types” and their molecular basis. It was agreed upon to retain the Sillence classification as the prototypic and universally accepted way to classify the degree of severity in OI; and to free the Sillence classification from any direct molecular reference. Thus, the many genes that may cause osteogenesis imperfecta have been listed separately. The proliferation of “OI types” to reflect each gene separately, advocated by some scholars, is more confusing than helpful in clinical practice.

Group 26 has seen the identification of several novel molecular mechanisms leading to hypophosphatemic rickets.

In Group 29 (Disorganized Development of Skeletal Components), neurofibromatosis type 1 has been included following the points made by Stevenson and others that although the main clinical features of NF1 are neurologic and cutaneous, the skeletal features are frequent, diagnostically helpful and clinically relevant [Stevenson et al., 2007].

Groups 30 (Overgrowth syndromes with significant skeletal involvement) and Group 31 (Genetic inflammatory/rheumatoid-like osteoarthropathies) have been newly added. Group 30 comprises disorders that present as overgrowth syndromes and have a significant skeletal component that is part of the diagnostic criteria for a specific condition. One condition has been tentatively included because of its conspicuous skeletal features [Nishimura et al., 2004; Schmidt et al., 2007]; however this condition remains incompletely delineated. Group 31 includes disorders with features of inflammation and skeletal involvement. The creation of these two groups has been suggested by the frequent diagnostic overlap between these disorders and primary skeletal disorders as well as by the identification of the genetic basis of such disorders in recent years, allowing for a more precise delineation of the phenotypes.

Finally, groups 32–40 are dedicated to the dysostoses and follow again anatomical criteria (cranium, face, axial skeleton, extremities) with additional criteria reflecting principles of embryonic development such as limb reduction or hypoplasia (proximal-distal growth) versus terminal differentiation and patterning of the digits or joint formation. These groups have seen a marked increase in conditions with identified molecular bases and there are indications of a much larger heterogeneity yet.

A single group, the Brachyolmias (formerly group 13), has been deleted. Following the inclusion of dominant brachyolmia in the TRPV4 group, the few remaining short-trunk disorders have been incorporated in the SED group.

## DISCUSSION

### Why “Groups”?

The assignment of individual disorders into groups has been practiced since the first versions of the “Nomenclature.” At that time, with little biochemical or molecular information available, the grouping of disorders reflected the belief that disorders with similar phenotypic features (e.g., *dysostosis multiplex*) might be caused by disturbances in related metabolic pathways or gene networks (in the case of dysostosis multiplex, lysosomal degradation). This notion has been confirmed by the identification of biochemically related groups, such as those of mineralization disorders or lysosomal disorders, and of genetic families such as the collagen 2 family, the FGFR3 family, and the DTDST family. The grouping of disorders is necessary because of the sheer number of conditions included, and can be helpful in making a differential diagnosis based on the main phenotypic findings, for example, in the mesomelic dysplasias or in chondrodysplasia punctata. Some groups are still defined by common radiographic features or by anatomical site involved. Moreover, the nosology committee recognizes that some readers may disagree with our placement of a clinical entity into one group, when it may fit equally well in another group.

### Which Classification Criteria to Use?

Criticism to the previous versions of the Nosology has focused on its “hybrid” nature, in the sense that it does not stick to a single systematic approach, be it clinical or molecular. This hybrid nature is intrinsic to the process of unraveling the underlying bases of skeletal diseases; disorders are classified on phenotypic similarities first, and as their molecular bases become understood they may be reclassified based on the gene or pathway that is abnormal. The first aim of the Nosology is to provide a reference list, and only secondarily to help in the diagnostic process. It must therefore coexist with other classifications that are based either on the clinical and radiographic approach to diagnosis, or the affected molecular systems and pathways. As more and more resources are published on the World Wide Web, crosslinking between classifications and databases may facilitate their simultaneous use.

Although care has been given to apply the inclusion criteria uniformly, there are disorders without proven molecular or biochemical defect for which inclusion in the Nosology as distinct entities seem somewhat arbitrary. For these disorders, discussion within the Nosology group, where individual opinions can be harmonized and, if needed, corrected by the collective expertise, is of great importance. Moreover, there are disorders listed in MIM that have not met our inclusion criteria, in most instances because of too few observations or because of the lack of features allowing clear diagnostic distinction from other disorders. It is likely that additional observations or the demonstration of a distinct molecular basis will allow for the inclusion of many of these disorders in the future, either as separate entities or as “variants” of already existing ones.

### Dysplasias Versus Dysostoses

Dysostoses are disorders affecting individual bones or group of bones. In contrast to the “dysplasias,” that arise frequently from defects in structural proteins, metabolic processes or in growth plate regulation, the dysostoses often arise from embryonic morphogenic defects and are thus more closely related to multiple malformation syndromes. Since the first inclusion of dysostoses in the 2001 revision, the number of “dysostoses” included in the Nosology has grown significantly. The present revision includes an even larger number of dysostoses reflecting the advances made in identifying their molecular basis. The boundaries between skeletal dysplasias and dysostoses, metabolic and molecular disorders, and multiple congenital anomalies syndromes is becoming progressively less sharp, and the diagnostic process requires knowledge that crosses between these subspecialty areas; the group of (cranio-)frontonasal disorders and the Franck–ter Haar syndrome can be cited as examples. The MIM catalogue contains many more entries, such as multiple malformation syndromes, that have some degree of skeletal involvement. Emphasis has been given to syndromes in which the skeletal component is prominent and/or essential to the diagnosis.

### OMIM and the Nosology

Because of the importance of consistency between parallel databases, the relationship between the Nosology and the OMIM database has been reviewed. The more comprehensive nature of the data collection and filing in MIM and the different nature of its revision process can lead to a divergence between the inclusion of nosologic entities and their denomination. Thus, MIM is in general more appositional, while the Nosology tries to do some “housekeeping” of entities by regrouping them and by eliminating those that have been incorporated into others. Efforts to made to harmonize the MIM and the Nosology are underway.

### Outlook

The increasing availability of massive parallel sequencing and other new sequencing technologies will likely result in a rapid identification of novel disease-causing genes, but also in novel phenotypes associated with mutations in genes already linked to other phenotypes. In the near future, the catalog of skeletal phenotypes with a genetic basis may become so large as to surpass the scope of a “Nosology” as we understand it presently, and the Nosology will transform into an annotated database.

Even in that case, the many revisions of the Nosology will hopefully have paved the way by setting standards for the recognition and definition of skeletal phenotypes. Past versions of the Nosology have been translated in different languages and have found their way into textbooks of pediatrics and genetics. At present, the Nosology may help the clinician who is struggling for a diagnosis, by providing a simple listing of disorders grouped by cardinal features. The Nosology offers a quick reminder of the many differential diagnoses for one given disorder. As an expert-reviewed list of currently recognized disorders, the Nosology also constitutes a standard against which a possible “new” disorder should be compared. Finally, the Nosology offers a catalogue of genes involved in skeletal development and homeostasis that will be of interest and of inspiration to all those who are working in skeletal biology and medicine.
